# Ethyl 3-(2-eth­oxy-2-oxoeth­oxy)-6-(tri­fluoro­meth­yl)furo[3,2-*c*]quinoline-2-carboxyl­ate

**DOI:** 10.1107/S1600536812046843

**Published:** 2012-11-24

**Authors:** B. Garudachari, Arun M. Islor, A. M. Vijesh, Thomas Gerber, Eric Hosten, Richard Betz

**Affiliations:** a National Institute of Technology-Karnataka, Department of Chemistry, Medicinal Chemistry Laboratory, Surathkal, Mangalore 575 025, India; b GITAM University, Department of Engineering Chemistry, GIT, Rushikonda, Visakhapatnam, A.P. 530 045, India; c Nelson Mandela Metropolitan University, Summerstrand Campus, Department of Chemistry, University Way, Summerstrand, PO Box 77000, Port Elizabeth, 6031, South Africa

## Abstract

In the title compound, C_19_H_16_F_3_NO_6_, a quinoline derivative featuring an annealated furan substituent, the mean planes of the carb­oxy substituents are at an angle of 74.3 (2)°. In the crystal, C—H⋯O contacts result in undulating chains along [110]. C—H⋯F contacts also occur. The shortest centroid–centroid distance between rings is 3.3376 (7) Å, involving two furan rings of neighbouring mol­ecules.

## Related literature

For background to the pharmacological activity of heterocyclic compounds, see: Isloor *et al.* (2000 ▶, 2009 ▶); Caprio *et al.* (2000 ▶); Kaur *et al.* (2010 ▶); Chou *et al.* (2010 ▶); Chen *et al.* (2004 ▶); Garudachari *et al.* (2012 ▶); Shingalapur *et al.* (2009 ▶). For graph-set analysis of hydrogen bonds, see: Etter *et al.* (1990 ▶); Bernstein *et al.* (1995 ▶).
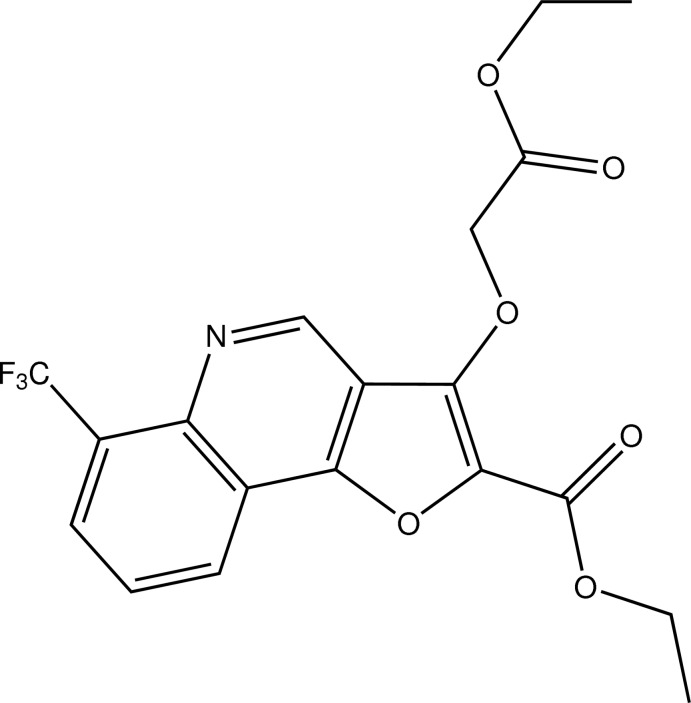


## Experimental

### 

#### Crystal data

C_19_H_16_F_3_NO_6_
*M* *_r_* = 411.33
Triclinic, 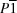

*a* = 8.9167 (3) Å
*b* = 8.9223 (3) Å
*c* = 13.4125 (5) Å
α = 102.895 (1)°
β = 97.098 (2)°
γ = 116.035 (1)°
*V* = 904.22 (5) Å^3^
*Z* = 2
Mo *K*α radiation
μ = 0.13 mm^−1^
*T* = 200 K
0.56 × 0.38 × 0.15 mm

#### Data collection

Bruker APEXII CCD diffractometer
Absorption correction: multi-scan (*SADABS*; Bruker, 2008 ▶) *T*_min_ = 0.918, *T*_max_ = 0.980
19743 measured reflections
4499 independent reflections
4019 reflections with *I* > 2σ(*I*)
*R*_int_ = 0.014

#### Refinement

*R*[*F* ^2^ > 2σ(*F* ^2^)] = 0.034
*wR*(*F* ^2^) = 0.096
*S* = 1.04
4499 reflections
264 parameters
H-atom parameters constrained
Δρ_max_ = 0.33 e Å^−3^
Δρ_min_ = −0.21 e Å^−3^

### 

Data collection: *APEX2* (Bruker, 2010 ▶); cell refinement: *SAINT* (Bruker, 2010 ▶); data reduction: *SAINT*; program(s) used to solve structure: *SHELXS97* (Sheldrick, 2008 ▶); program(s) used to refine structure: *SHELXL97* (Sheldrick, 2008 ▶); molecular graphics: *ORTEP-3* (Farrugia, 2012 ▶) and *Mercury* (Macrae *et al.*, 2008 ▶); software used to prepare material for publication: *SHELXL97* and *PLATON* (Spek, 2009 ▶).

## Supplementary Material

Crystal structure: contains datablock(s) I, global. DOI: 10.1107/S1600536812046843/fj2605sup1.cif

Supplementary material file. DOI: 10.1107/S1600536812046843/fj2605Isup2.cdx

Structure factors: contains datablock(s) I. DOI: 10.1107/S1600536812046843/fj2605Isup3.hkl

Supplementary material file. DOI: 10.1107/S1600536812046843/fj2605Isup4.cml

Additional supplementary materials:  crystallographic information; 3D view; checkCIF report

## Figures and Tables

**Table 1:** Hydrogen-bond geometry (Å, °)

*D*—H⋯*A*	*D*—H	H⋯*A*	*D*⋯*A*	*D*—H⋯*A*
C5—H5⋯O3^i^	0.95	2.44	3.2556 (13)	144
C13—H13*B*⋯F1^ii^	0.99	2.46	3.2829 (12)	141

Symmetry codes: (i) 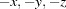; (ii) 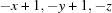.

**Fig. 1.**
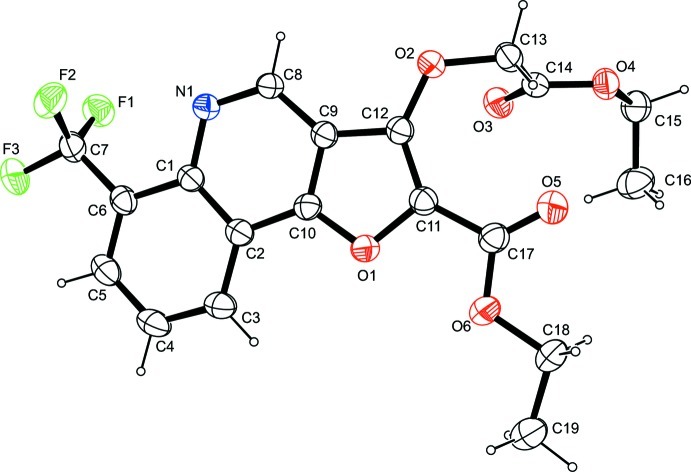
The molecular structure of the title compound, with atom labels and anisotropic displacement ellipsoids (drawn at 50% probability level).

**Fig. 2.**
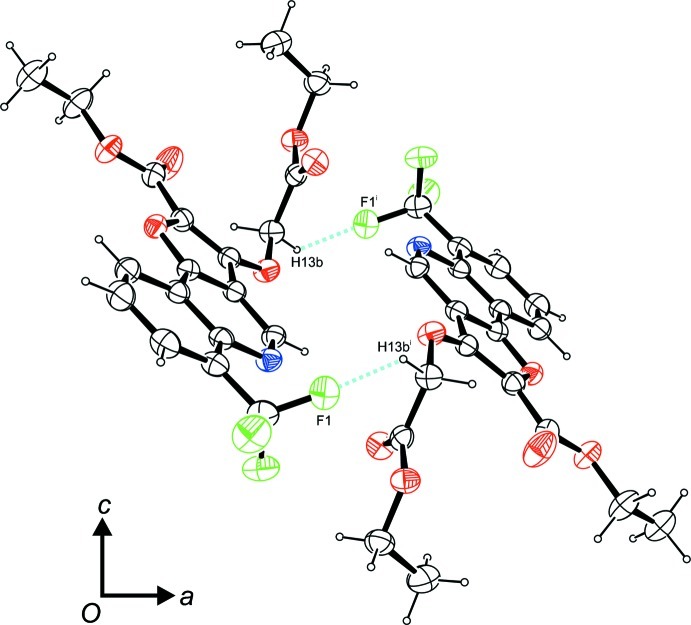
Intermolecular contacts, viewed along [0 1 0]. For clarity, only the C–H···F contacts are depicted. Symmetry operator: i -x + 1, -y + 1, -z.

## supplementary crystallographic information

### Comment

Heterocyclic compounds play an important role in our ongoing interest in developing new antimicrobial agents (Isloor *et al.*, 2000; Isloor *et al.*, 2009). The quinoline nucleus is one of the most important and widely exploited heterocyclic ring systems for the development of bioactive molecules (Caprio *et al.*, 2000). Members of this family have a wide range of applications in pharmacology as antimalarial (Kaur *et al.*, 2010), antitumor (Chou *et al.*, 2010), anticancer (Chen *et al.*, 2004), antimicrobial (Garudachari *et al.*, 2012) and antiviral (Shingalapur *et al.*, 2009) agents. In view of the promising biological and pharmaceutical activity, the title compound was synthesized to study its crystal structure.

The aromatic scaffold is essentially flat. The least-squares plane defined by all the non-hydrogen atoms (r.m.s. of all fitted atoms = 0.0175 Å) features the carbon atom bearing the trifluoromethyl group as the atom deviating most from this common plane by 0.029 (1) Å. The least-squares planes defined by the respective atoms of the phenyl moiety and the furane moiety intersect at an angle of 2.26 (6) ° while they enclose angles of 0.76 (5) ° and 1.51 (6) ° with the least-squares plane defined by the atoms of the central heterocycle. The fluorine atoms of the trifluoromethyl group adopt a staggered conformation with respect to the nitrogen atom. The two carboxy substituents bonded to the furane system are orientated nearly perpendicular to each other with the least-squares planes defined by their respective atoms enclosing an angle of 74.3 (2) °. (Fig. 1).

In the crystal, C–H···O and C–H···F contacts are apparent whose range invariably falls by more than 0.2 Å below the sum of van-der-Waals radii of the atoms participating. While the former contacts are supported by the hydrogen atom in *ortho* position to the trifluoromethyl group as the donor and one of the double bonded oxygen atoms as the acceptor, the C–H···F contacts stem from one of the hydrogen atoms of a methylene group. Metrical parameters as well as information about the symmetry of these contacts are summarized in Table 1. In total, the molecules are connected to undulated chains along [1 1 0]. In terms of graph-set analysis (Etter *et al.*, 1990; Bernstein *et al.*, 1995), the descriptor for these contacts is *R*^2^_2_(22)*R*^2^_2_(24) on the unary level. The shortest intercentroid distance between two aromatic systems was measured at 3.3376 (7) Å and is apparent between two furane moieties in neighbouring molecules (Fig. 2).

### Experimental

To a suspension of ethyl 4-hydroxy-8-(trifluoromethyl)quinoline-3-carboxylate (1.0 g, 0.0035 mol) and potassium carbonate (1.06 g, 0.0077 mol) in dimethylformamide (10 ml) ethyl 4-chloroacetoacetate (0.943 g, 0.0077 mol) was added. The mixture was allowed to stir at 80 °C for 2 h and was then quenched by the slow addition of water (25 ml). The precipitated solids were collected by filtration and recrystallized from ethanol, yield: 1.20 g (83.33%).

### Refinement

Carbon-bound H atoms were placed in calculated positions (C–H 0.95 Å for aromatic carbon atoms and C–H 0.99 Å for methylene groups) and were included in the refinement in the riding model approximation, with *U*(H) set to 1.2*U*_eq_(C). The H atoms of the methyl groups were allowed to rotate with a fixed angle around the C–C bond to best fit the experimental electron density (HFIX 137 in the *SHELX* program suite (Sheldrick, 2008)), with *U*(H) set to 1.5*U*_eq_(C).

### Crystal data

**Table:**

C_19_H_16_F_3_NO_6_	*Z* = 2
*M**_r_* = 411.33	*F*(000) = 424
Triclinic, *P*1	*D*_x_ = 1.511 Mg m^−^^3^
Hall symbol: -P 1	Melting point = 400–398 K
*a* = 8.9167 (3) Å	Mo *K*α radiation, λ = 0.71073 Å
*b* = 8.9223 (3) Å	Cell parameters from 9936 reflections
*c* = 13.4125 (5) Å	θ = 2.7–28.5°
α = 102.895 (1)°	µ = 0.13 mm^−^^1^
β = 97.098 (2)°	*T* = 200 K
γ = 116.035 (1)°	Block, colourless
*V* = 904.22 (5) Å^3^	0.56 × 0.38 × 0.15 mm

### Data collection

**Table:**

Bruker APEXII CCD diffractometer	4499 independent reflections
Radiation source: fine-focus sealed tube	4019 reflections with *I* > 2σ(*I*)
Graphite monochromator	*R*_int_ = 0.014
φ and ω scans	θ_max_ = 28.5°, θ_min_ = 2.7°
Absorption correction: multi-scan (*SADABS*; Bruker, 2008)	*h* = −11→11
*T*_min_ = 0.918, *T*_max_ = 0.980	*k* = −10→11
19743 measured reflections	*l* = −17→17

### Refinement

**Table:**

Refinement on *F*^2^	Primary atom site location: structure-invariant direct methods
Least-squares matrix: full	Secondary atom site location: difference Fourier map
*R*[*F*^2^ > 2σ(*F*^2^)] = 0.034	Hydrogen site location: inferred from neighbouring sites
*wR*(*F*^2^) = 0.096	H-atom parameters constrained
*S* = 1.04	*w* = 1/[σ^2^(*F*_o_^2^) + (0.0475*P*)^2^ + 0.2384*P*] where *P* = (*F*_o_^2^ + 2*F*_c_^2^)/3
4499 reflections	(Δ/σ)_max_ < 0.001
264 parameters	Δρ_max_ = 0.33 e Å^−^^3^
0 restraints	Δρ_min_ = −0.21 e Å^−^^3^

### Fractional atomic coordinates and isotropic or equivalent isotropic displacement parameters (Å^2^)

**Table:**

	*x*	*y*	*z*	*U*_iso_*/*U*_eq_	
F1	0.11127 (9)	−0.15513 (9)	−0.26381 (6)	0.04187 (17)	
F2	−0.02108 (11)	−0.07488 (11)	−0.36753 (6)	0.04779 (19)	
F3	−0.14712 (10)	−0.33572 (10)	−0.35590 (6)	0.0530 (2)	
O1	−0.01551 (9)	0.36022 (10)	0.09774 (6)	0.02896 (16)	
O2	0.39515 (10)	0.69558 (10)	0.08994 (6)	0.03272 (17)	
O3	0.52925 (10)	0.64136 (10)	0.26519 (6)	0.03546 (18)	
O4	0.61251 (10)	0.91813 (10)	0.36366 (6)	0.03402 (17)	
O5	0.21017 (12)	0.77916 (13)	0.29209 (8)	0.0532 (3)	
O6	−0.03854 (11)	0.53320 (11)	0.26364 (6)	0.03750 (19)	
N1	0.14170 (11)	0.17822 (11)	−0.15495 (7)	0.02913 (18)	
C1	−0.01414 (12)	0.06686 (13)	−0.14013 (8)	0.0267 (2)	
C2	−0.08456 (13)	0.11463 (13)	−0.05685 (8)	0.0274 (2)	
C3	−0.24555 (14)	−0.00514 (15)	−0.04615 (9)	0.0329 (2)	
H3	−0.2910	0.0290	0.0098	0.040*	
C4	−0.33531 (14)	−0.17029 (15)	−0.11687 (10)	0.0375 (2)	
H4	−0.4439	−0.2511	−0.1102	0.045*	
C5	−0.26790 (14)	−0.22145 (14)	−0.19950 (9)	0.0352 (2)	
H5	−0.3314	−0.3369	−0.2480	0.042*	
C6	−0.11170 (13)	−0.10699 (13)	−0.21111 (8)	0.0300 (2)	
C7	−0.04134 (15)	−0.16644 (14)	−0.29876 (9)	0.0355 (2)	
C8	0.23213 (13)	0.33840 (13)	−0.08870 (8)	0.0278 (2)	
H8	0.3388	0.4156	−0.0994	0.033*	
C9	0.17610 (12)	0.39984 (13)	−0.00194 (7)	0.02619 (19)	
C10	0.02059 (12)	0.28672 (13)	0.01161 (7)	0.02626 (19)	
C11	0.12332 (13)	0.53029 (13)	0.14338 (8)	0.0285 (2)	
C12	0.24320 (13)	0.55879 (13)	0.08426 (8)	0.0270 (2)	
C13	0.48632 (14)	0.84001 (13)	0.18518 (8)	0.0311 (2)	
H13A	0.4109	0.8901	0.2052	0.037*	
H13B	0.5887	0.9326	0.1728	0.037*	
C14	0.54340 (13)	0.78448 (13)	0.27476 (8)	0.0285 (2)	
C15	0.67733 (15)	0.88722 (16)	0.45808 (9)	0.0390 (3)	
H15A	0.7590	1.0013	0.5120	0.047*	
H15B	0.7409	0.8220	0.4400	0.047*	
C16	0.53376 (19)	0.7847 (2)	0.50341 (11)	0.0505 (3)	
H16A	0.4600	0.6665	0.4534	0.076*	
H16B	0.4650	0.8444	0.5162	0.076*	
H16C	0.5826	0.7762	0.5702	0.076*	
C17	0.10712 (14)	0.63031 (14)	0.24067 (9)	0.0321 (2)	
C18	−0.06969 (18)	0.61382 (17)	0.35973 (10)	0.0453 (3)	
H18A	−0.0846	0.7152	0.3533	0.054*	
H18B	0.0287	0.6556	0.4209	0.054*	
C19	−0.23018 (19)	0.47652 (19)	0.37435 (11)	0.0494 (3)	
H19A	−0.3256	0.4339	0.3123	0.074*	
H19B	−0.2579	0.5268	0.4376	0.074*	
H19C	−0.2125	0.3787	0.3827	0.074*	

### Atomic displacement parameters (Å^2^)

**Table:**

	*U*^11^	*U*^22^	*U*^33^	*U*^12^	*U*^13^	*U*^23^
F1	0.0383 (4)	0.0366 (4)	0.0466 (4)	0.0170 (3)	0.0099 (3)	0.0082 (3)
F2	0.0582 (5)	0.0521 (4)	0.0312 (3)	0.0230 (4)	0.0127 (3)	0.0165 (3)
F3	0.0485 (4)	0.0340 (4)	0.0471 (4)	0.0056 (3)	0.0067 (3)	−0.0071 (3)
O1	0.0267 (3)	0.0310 (4)	0.0284 (3)	0.0128 (3)	0.0085 (3)	0.0095 (3)
O2	0.0293 (4)	0.0297 (4)	0.0281 (4)	0.0060 (3)	0.0072 (3)	0.0066 (3)
O3	0.0373 (4)	0.0289 (4)	0.0383 (4)	0.0147 (3)	0.0084 (3)	0.0102 (3)
O4	0.0367 (4)	0.0296 (4)	0.0287 (4)	0.0124 (3)	0.0053 (3)	0.0054 (3)
O5	0.0430 (5)	0.0424 (5)	0.0519 (5)	0.0087 (4)	0.0200 (4)	−0.0047 (4)
O6	0.0389 (4)	0.0354 (4)	0.0370 (4)	0.0163 (3)	0.0186 (3)	0.0079 (3)
N1	0.0281 (4)	0.0280 (4)	0.0265 (4)	0.0092 (3)	0.0070 (3)	0.0089 (3)
C1	0.0255 (4)	0.0264 (4)	0.0255 (4)	0.0095 (4)	0.0036 (4)	0.0110 (4)
C2	0.0252 (4)	0.0287 (5)	0.0271 (5)	0.0108 (4)	0.0044 (4)	0.0121 (4)
C3	0.0274 (5)	0.0362 (5)	0.0352 (5)	0.0123 (4)	0.0092 (4)	0.0168 (4)
C4	0.0270 (5)	0.0346 (5)	0.0442 (6)	0.0066 (4)	0.0076 (4)	0.0186 (5)
C5	0.0309 (5)	0.0275 (5)	0.0371 (5)	0.0071 (4)	0.0014 (4)	0.0109 (4)
C6	0.0295 (5)	0.0276 (5)	0.0278 (5)	0.0103 (4)	0.0021 (4)	0.0095 (4)
C7	0.0357 (5)	0.0278 (5)	0.0312 (5)	0.0084 (4)	0.0040 (4)	0.0050 (4)
C8	0.0253 (4)	0.0282 (5)	0.0263 (4)	0.0092 (4)	0.0072 (4)	0.0095 (4)
C9	0.0253 (4)	0.0273 (4)	0.0243 (4)	0.0112 (4)	0.0041 (3)	0.0093 (4)
C10	0.0255 (4)	0.0304 (5)	0.0242 (4)	0.0135 (4)	0.0062 (3)	0.0110 (4)
C11	0.0269 (5)	0.0289 (5)	0.0285 (5)	0.0128 (4)	0.0059 (4)	0.0088 (4)
C12	0.0261 (4)	0.0279 (5)	0.0258 (4)	0.0121 (4)	0.0049 (4)	0.0092 (4)
C13	0.0303 (5)	0.0251 (5)	0.0305 (5)	0.0086 (4)	0.0052 (4)	0.0068 (4)
C14	0.0237 (4)	0.0270 (5)	0.0302 (5)	0.0087 (4)	0.0082 (4)	0.0075 (4)
C15	0.0365 (6)	0.0431 (6)	0.0296 (5)	0.0149 (5)	0.0037 (4)	0.0088 (4)
C16	0.0531 (8)	0.0612 (8)	0.0414 (7)	0.0262 (7)	0.0187 (6)	0.0230 (6)
C17	0.0314 (5)	0.0347 (5)	0.0329 (5)	0.0178 (4)	0.0100 (4)	0.0100 (4)
C18	0.0492 (7)	0.0425 (6)	0.0410 (6)	0.0199 (6)	0.0236 (5)	0.0055 (5)
C19	0.0513 (7)	0.0509 (7)	0.0476 (7)	0.0230 (6)	0.0267 (6)	0.0139 (6)

### Geometric parameters (Å, º)

**Table:**

F1—C7	1.3350 (14)	C5—H5	0.9500
F2—C7	1.3412 (13)	C6—C7	1.5011 (16)
F3—C7	1.3462 (12)	C8—C9	1.4155 (14)
O1—C10	1.3444 (12)	C8—H8	0.9500
O1—C11	1.3996 (12)	C9—C10	1.3748 (13)
O2—C12	1.3463 (12)	C9—C12	1.4347 (14)
O2—C13	1.4291 (12)	C11—C12	1.3772 (14)
O3—C14	1.2019 (13)	C11—C17	1.4698 (14)
O4—C14	1.3275 (12)	C13—C14	1.5113 (15)
O4—C15	1.4591 (14)	C13—H13A	0.9900
O5—C17	1.2029 (14)	C13—H13B	0.9900
O6—C17	1.3277 (13)	C15—C16	1.5028 (18)
O6—C18	1.4511 (13)	C15—H15A	0.9900
N1—C8	1.3140 (13)	C15—H15B	0.9900
N1—C1	1.3791 (13)	C16—H16A	0.9800
C1—C2	1.4211 (14)	C16—H16B	0.9800
C1—C6	1.4215 (14)	C16—H16C	0.9800
C2—C10	1.4050 (14)	C18—C19	1.4915 (18)
C2—C3	1.4128 (14)	C18—H18A	0.9900
C3—C4	1.3655 (16)	C18—H18B	0.9900
C3—H3	0.9500	C19—H19A	0.9800
C4—C5	1.4063 (17)	C19—H19B	0.9800
C4—H4	0.9500	C19—H19C	0.9800
C5—C6	1.3722 (15)		
			
C10—O1—C11	106.31 (8)	O1—C11—C17	113.88 (9)
C12—O2—C13	120.56 (8)	O2—C12—C11	134.90 (10)
C14—O4—C15	116.50 (9)	O2—C12—C9	118.69 (9)
C17—O6—C18	116.38 (9)	C11—C12—C9	106.39 (9)
C8—N1—C1	118.85 (9)	O2—C13—C14	111.56 (8)
N1—C1—C2	123.90 (9)	O2—C13—H13A	109.3
N1—C1—C6	119.02 (9)	C14—C13—H13A	109.3
C2—C1—C6	117.07 (9)	O2—C13—H13B	109.3
C10—C2—C3	124.76 (10)	C14—C13—H13B	109.3
C10—C2—C1	113.96 (9)	H13A—C13—H13B	108.0
C3—C2—C1	121.27 (9)	O3—C14—O4	126.09 (10)
C4—C3—C2	119.53 (10)	O3—C14—C13	124.44 (9)
C4—C3—H3	120.2	O4—C14—C13	109.47 (9)
C2—C3—H3	120.2	O4—C15—C16	111.75 (10)
C3—C4—C5	120.36 (10)	O4—C15—H15A	109.3
C3—C4—H4	119.8	C16—C15—H15A	109.3
C5—C4—H4	119.8	O4—C15—H15B	109.3
C6—C5—C4	120.94 (10)	C16—C15—H15B	109.3
C6—C5—H5	119.5	H15A—C15—H15B	107.9
C4—C5—H5	119.5	C15—C16—H16A	109.5
C5—C6—C1	120.81 (10)	C15—C16—H16B	109.5
C5—C6—C7	119.75 (10)	H16A—C16—H16B	109.5
C1—C6—C7	119.43 (9)	C15—C16—H16C	109.5
F1—C7—F2	106.89 (9)	H16A—C16—H16C	109.5
F1—C7—F3	105.90 (9)	H16B—C16—H16C	109.5
F2—C7—F3	106.11 (9)	O5—C17—O6	124.62 (10)
F1—C7—C6	112.95 (9)	O5—C17—C11	125.11 (10)
F2—C7—C6	112.93 (10)	O6—C17—C11	110.27 (9)
F3—C7—C6	111.55 (9)	O6—C18—C19	106.84 (10)
N1—C8—C9	122.26 (9)	O6—C18—H18A	110.4
N1—C8—H8	118.9	C19—C18—H18A	110.4
C9—C8—H8	118.9	O6—C18—H18B	110.4
C10—C9—C8	118.01 (9)	C19—C18—H18B	110.4
C10—C9—C12	105.74 (9)	H18A—C18—H18B	108.6
C8—C9—C12	136.23 (9)	C18—C19—H19A	109.5
O1—C10—C9	111.91 (9)	C18—C19—H19B	109.5
O1—C10—C2	125.07 (9)	H19A—C19—H19B	109.5
C9—C10—C2	123.01 (9)	C18—C19—H19C	109.5
C12—C11—O1	109.64 (9)	H19A—C19—H19C	109.5
C12—C11—C17	136.46 (10)	H19B—C19—H19C	109.5
			
C8—N1—C1—C2	0.10 (15)	C12—C9—C10—C2	−179.09 (9)
C8—N1—C1—C6	−179.49 (9)	C3—C2—C10—O1	1.34 (16)
N1—C1—C2—C10	−1.04 (14)	C1—C2—C10—O1	−177.96 (9)
C6—C1—C2—C10	178.55 (8)	C3—C2—C10—C9	−179.52 (9)
N1—C1—C2—C3	179.63 (9)	C1—C2—C10—C9	1.18 (14)
C6—C1—C2—C3	−0.77 (14)	C10—O1—C11—C12	0.00 (11)
C10—C2—C3—C4	−179.03 (10)	C10—O1—C11—C17	−179.01 (8)
C1—C2—C3—C4	0.21 (15)	C13—O2—C12—C11	16.43 (17)
C2—C3—C4—C5	0.33 (16)	C13—O2—C12—C9	−165.38 (9)
C3—C4—C5—C6	−0.28 (17)	O1—C11—C12—O2	178.43 (10)
C4—C5—C6—C1	−0.32 (16)	C17—C11—C12—O2	−2.9 (2)
C4—C5—C6—C7	178.76 (10)	O1—C11—C12—C9	0.08 (11)
N1—C1—C6—C5	−179.56 (9)	C17—C11—C12—C9	178.78 (12)
C2—C1—C6—C5	0.82 (15)	C10—C9—C12—O2	−178.80 (8)
N1—C1—C6—C7	1.35 (14)	C8—C9—C12—O2	2.89 (17)
C2—C1—C6—C7	−178.26 (9)	C10—C9—C12—C11	−0.14 (11)
C5—C6—C7—F1	−120.41 (11)	C8—C9—C12—C11	−178.44 (11)
C1—C6—C7—F1	58.69 (13)	C12—O2—C13—C14	64.30 (12)
C5—C6—C7—F2	118.14 (11)	C15—O4—C14—O3	0.15 (15)
C1—C6—C7—F2	−62.76 (13)	C15—O4—C14—C13	−178.99 (9)
C5—C6—C7—F3	−1.26 (15)	O2—C13—C14—O3	6.79 (14)
C1—C6—C7—F3	177.84 (9)	O2—C13—C14—O4	−174.05 (8)
C1—N1—C8—C9	0.79 (15)	C14—O4—C15—C16	−78.05 (13)
N1—C8—C9—C10	−0.65 (15)	C18—O6—C17—O5	−1.97 (18)
N1—C8—C9—C12	177.51 (10)	C18—O6—C17—C11	178.42 (10)
C11—O1—C10—C9	−0.10 (11)	C12—C11—C17—O5	2.2 (2)
C11—O1—C10—C2	179.13 (9)	O1—C11—C17—O5	−179.15 (11)
C8—C9—C10—O1	178.82 (8)	C12—C11—C17—O6	−178.20 (11)
C12—C9—C10—O1	0.15 (11)	O1—C11—C17—O6	0.45 (13)
C8—C9—C10—C2	−0.42 (14)	C17—O6—C18—C19	−174.85 (11)

### Hydrogen-bond geometry (Å, º)

**Table:**

*D*—H···*A*	*D*—H	H···*A*	*D*···*A*	*D*—H···*A*
C5—H5···O3^i^	0.95	2.44	3.2556 (13)	144
C13—H13*B*···F1^ii^	0.99	2.46	3.2829 (12)	141

Symmetry codes: (i) −*x*, −*y*, −*z*; (ii) −*x*+1, −*y*+1, −*z*.

## References

[bb1] Bernstein, J., Davis, R. E., Shimoni, L. & Chang, N.-L. (1995). *Angew. Chem. Int. Ed. Engl.* **34**, 1555–1573.

[bb2] Bruker (2008). *SADABS* Bruker Inc., Madison, Wisconsin, USA.

[bb3] Bruker (2010). *APEX2* and *SAINT* Bruker AXS Inc., Madison, Wisconsin, USA.

[bb4] Caprio, V., Guyen, B., Opoku-Boahen, Y., Mann, J., Gowan, S. M., Kelland, L. M., Read, M. A. & Neidle, S. (2000). *Bioorg. Med. Chem. Lett.* **10**, 2063–2066. 10.1016/s0960-894x(00)00378-4 10999471

[bb5] Chen, Y. L., Hung, H. M., Lu, C. M., Li, K. C. & Tzeng, C. C. (2004). *Bioorg. Med. Chem.* **12**, 6539–6546. 10.1016/j.bmc.2004.09.025 15556770

[bb6] Chou, L. C., Tsai, M. T., Hsu, M. H., Wang, S. H., Way, T. D., Huang, C. H., Lin, H. Y., Qian, K., Dong, Y., Lee, K. H., Huang, L. J. & Kuo, S. C. (2010). *J. Med. Chem.* **53**, 8047–8058. 10.1021/jm100780c 20973552

[bb7] Etter, M. C., MacDonald, J. C. & Bernstein, J. (1990). *Acta Cryst.* B**46**, 256–262. 10.1107/s0108768189012929 2344397

[bb8] Farrugia, L. J. (2012). *J. Appl. Cryst.* **45**, 849–854.

[bb9] Garudachari, B., Satyanarayana, M. N., Thippeswamy, B., Shivakumar, C. K., Shivananda, K. N. & Isloor, A. M. (2012). *Eur. J. Med. Chem.* **54**, 900–906. 10.1016/j.ejmech.2012.05.027 22732060

[bb10] Isloor, A. M., Kalluraya, B. & Rao, M. (2000). *J. Saudi Chem. Soc.* **4**, 265–270.

[bb11] Isloor, A. M., Kalluraya, B. & Shetty, P. (2009). *Eur. J. Med. Chem.* **44**, 3784–3787. 10.1016/j.ejmech.2009.04.038 19464087

[bb12] Kaur, K., Jain, M., Reddy, R. P. & Jain, R. (2010). *Eur. J. Med. Chem.* **45**, 3245–3264. 10.1016/j.ejmech.2010.04.011 20466465

[bb13] Macrae, C. F., Bruno, I. J., Chisholm, J. A., Edgington, P. R., McCabe, P., Pidcock, E., Rodriguez-Monge, L., Taylor, R., van de Streek, J. & Wood, P. A. (2008). *J. Appl. Cryst.* **41**, 466–470.

[bb14] Sheldrick, G. M. (2008). *Acta Cryst.* A**64**, 112–122. 10.1107/S0108767307043930 18156677

[bb15] Shingalapur, R. V., Hosamani, K. M. & Keri, R. S. (2009). *Eur. J. Med. Chem.* **44**, 4244–4248. 10.1016/j.ejmech.2009.05.021 19540630

[bb16] Spek, A. L. (2009). *Acta Cryst.* D**65**, 148–155. 10.1107/S090744490804362X PMC2631630 19171970

